# Prevalence of Malnutrition, Frailty, and Cachexia Across Different Cancer Subtypes: Distribution by Age (<65 and ≥65)

**DOI:** 10.3390/jcm15093202

**Published:** 2026-04-22

**Authors:** Tanju Kapagan, Cagla Ecem Kılıc, Beyza Arslansoy, Ece Yontan, Zozan Ozcalimli, Beyza Canan Ozkan Kardes, Mehmet Turkmencalıkoglu, Esma Yetim, Nilufer Bulut, Gokmen Umut Erdem

**Affiliations:** 1 Department of Medical Oncology, Basaksehir Cam and Sakura City Hospital, University of Health Sciences, 34480 Istanbul, Turkey; esmayetim91@gmail.com (E.Y.); ferlut@gmail.com (N.B.); gokmenumut@hotmail.com (G.U.E.); 2 Department of Internal Medicine, Basaksehir Cam and Sakura City Hospital, University of Health Sciences, 34480 Istanbul, Turkey; caglaecem3895@hotmail.com (C.E.K.); beyza_arslansoy@hotmail.com (B.A.); eceyontan2@gmail.com (E.Y.); zzn.ozcalimli@gmail.com (Z.O.); cananozkan96@gmail.com (B.C.O.K.); mehmetturkmencalik@gmail.com (M.T.)

**Keywords:** neoplasms, malnutrition, frailty, cachexia, aging

## Abstract

**Background:** The aim of this study is to determine the prevalence of malnutrition, frailty, and cachexia in patients with different cancer subtypes undergoing oncological treatment using various screening scales and to evaluate the distribution of these conditions by geriatric age groups. **Methods:** This cross-sectional study included 4595 patients with different cancer subtypes undergoing oncological treatment. Nutritional status was assessed using the Mini Nutritional Assessment—Short (MNA-S) form; frailty was evaluated with the Fatigue, Resistance, Ambulation, Illnesses, and Loss of weight (FRAIL) scale; and cachexia was determined based on >5% weight loss in the last 6 months or a body mass index (BMI) < 20 kg/m^2^, together with an additional >2% weight loss. Patients were divided into two groups by geriatric age. **Results:** A total of 36% of the patients included in the study were geriatric (≥65 years old). The prevalence of malnutrition, frailty, and cachexia in the general population was determined to be 40%, 45%, and 44%, respectively. All three conditions were found to be statistically significantly more prevalent in the geriatric age group (*p* < 0.001 for all comparisons). **Conclusions:** It was determined that the prevalence of malnutrition, frailty, and cachexia was high in cancer patients receiving systemic oncological treatments including chemotherapy, hormonal therapy, targeted therapy, and immunotherapy; these syndromes are significantly more common, particularly in the geriatric patient group.

## 1. Introduction

Malnutrition is characterized by loss of body mass and function due to an imbalance of energy and nutrients [1]. Frailty refers to increased sensitivity to stress factors, particularly during the aging process, as a result of decreased physiological reserves [2], while cachexia is a syndrome of resistant muscle loss accompanied by inflammation and metabolic disorders [3].

Cancer disrupts metabolic balance, leading to the development of catabolic complications such as malnutrition, frailty, and cachexia; this reduces treatment tolerance and increases mortality. While chemotherapy, radiotherapy, and targeted agents are effective in controlling tumors, they can have adverse effects on nutritional status and muscle mass [4]. Treatment-related loss of appetite, nausea, mucositis, taste changes, and gastrointestinal side effects reduce energy intake, accelerating malnutrition and muscle loss [5].

These conditions are biologically interconnected in cancer patients, as systemic inflammation, metabolic dysregulation, and reduced nutritional intake collectively contribute to muscle loss, functional decline, and progressive catabolism, leading to a significant overlap between malnutrition, frailty, and cachexia [6]. Although there are numerous studies on each of these syndromes in the literature, the number of studies that evaluate them together and address their distribution by age is limited, which limits a comprehensive understanding of their clinical overlap [7,8].

This study aimed to determine the prevalence of malnutrition, frailty, and cachexia in a large group of cancer patients and to examine their age-related distribution in particular.

## 2. Methods

### 2.1. Research Ethics

The protocol for sample collection was approved by the Basaksehir Cam and Sakura City Hospital Ethics Committee and was carried out according to the principles of the Declaration of Helsinki.

### 2.2. Study Population

This study was conducted as a single-center, cross-sectional study. The study included a total of 4595 adult cancer patients with histopathologically confirmed malignancy who were undergoing active treatment in the chemotherapy unit. The data were collected between June 2022 and August 2024. The questionnaires and all assessments, including the MNA-SF and FRAIL scales, were administered through face-to-face interviews to the patients by the nurses and physicians working in the chemotherapy unit. Patients were divided into two groups (<65 and ≥65).

Missing data were handled using a complete-case analysis approach. Patient-reported variables, including weight history and questionnaire-based items, were collected from interviews and medical records when available, and cases with missing key data were excluded from analyses.

### 2.3. Scales

#### 2.3.1. Mini Nutritional Assessment—Short Form (MNA-SF) Was Used for Malnutrition Screening

Within this scale, the following parameters were assessed over a three-month period: nutritional intake, weight loss, mobility, psychological stress or acute illness, neuropsychological status, and body mass index (BMI). Food intake over the past three months (0 = severe decrease, 1 = moderate decrease, 2 = no decrease), weight loss over the past three months (0 = >3 kg, 1 = unknown, 2 = 1–3 kg, 3 = none), mobility (0 = bedridden/chair-bound, 1 = able to move within the home, 2 = able to go outside), psychological stress or acute illness within the past three months (0 = yes, 2 = no), neuropsychological status (0 = severe impairment, 1 = mild impairment, 2 = normal), and BMI (0 = <19, 1 = 19–<21, 2 = 21–<23, 3 = ≥23) were assessed, and the resulting total score was classified as 12–14 indicating normal nutritional status, 8–11 indicating risk of malnutrition, and 0–7 indicating malnutrition (Table 1).

#### 2.3.2. Fatigue, Resistance, Ambulation, Illnesses, and Loss of Weight (FRAIL) Scale Was Used to Assess Frailty

Fatigue, stair climbing, walking ability, presence of illness, and weight loss within a year were evaluated on this scale. Fatigue level over the past four weeks (0 = Sometimes, A little, or Never; 1 = All the time or Most of the time), difficulty climbing 10 steps without assistance (0 = No, 1 = Yes), difficulty walking without assistance (0 = No, 1 = Yes), presence of diseases (hypertension, diabetes, cancer [excluding minor skin cancer], chronic lung disease, myocardial infarction, congestive heart failure, angina, asthma, arthritis, stroke, and kidney disease) (0 = 0–4 diseases, 1 = 5–11 diseases), and weight change compared with one year earlier (0 = <5%, 1 = >5%) were evaluated. A total score of 0 was considered healthy, 1–2 points pre-frail, and 3–5 points frail (Table 2).

#### 2.3.3. The Following Criteria Were Used for the Assessment of Cachexia

Unintentional weight loss: Low BMI parameters in this scale were evaluated.

Cachexia was accepted when unintentional weight loss exceeded 5% in the last six months or when BMI was <20 kg/m^2^ and weight loss exceeded 2%.

The definition of cachexia used in this study was based on objective and widely applied clinical criteria [9], including unintentional weight loss and BMI thresholds, to ensure consistency and feasibility in this large cross-sectional clinical cohort.

### 2.4. Inclusion Criteria for Patients Were as Follows

Being 18 years of age or older; having a cancer diagnosis; and receiving active chemotherapy, hormonal therapy, targeted therapy, or immunotherapy due to the cancer diagnosis.

### 2.5. Exclusion Criteria for Patients Were as Follows

Having a diagnosis of a second primary malignancy; having completed treatment for cancer and being in the follow-up period; having an ECOG performance status of 4; and missing key data in variables dependent on patient recall (such as weight history and questionnaire-based items).

### 2.6. Statistical Analysis

Statistical analyses were performed using SPSS version 27.0. The Kolmogorov–Smirnov test was used to assess the normality of the age variable; since the distribution was normal, age was presented as mean ± standard deviation. The rates of malnutrition, frailty, and cachexia were expressed as numbers and percentages. Comparisons of malnutrition, frailty, and cachexia by geriatric age groups were performed using the chi-square or Fisher’s exact tests, as appropriate. A *p*-value of <0.05 was considered statistically significant.

## 3. Results

### 3.1. Distribution of Demographic and Clinical Variables

A total of 2313 (50.3%) of the 4595 patients were male and 2282 (49.7%) were female. The mean age was 58.9 ± 12.2 years, and 1672 patients (36.4%) were aged 65 years or older. The prevalence rates of malnutrition, frailty, and cachexia were determined to be 40% (n = 1850), 45% (n = 2052), and 44% (n = 2042), respectively. The distribution of participants’ demographic characteristics in relation to nutrition, frailty, and cachexia rates is shown in Table 3.

### 3.2. Patient Distribution by Diagnosis Type

Our study included a total of 912 lung cancer patients, 835 of whom had non-small cell lung carcinoma and 77 of whom had small cell lung carcinoma, with lung cancer constituting the largest patient group. Among colorectal cancers, there were a total of 900 patients, including 660 colon carcinomas and 240 rectal carcinomas. There were 841 patients in the breast cancer group. Details regarding the distribution of patients by cancer type are presented in Table 4.

### 3.3. Distribution of Malnutrition, Frailty, and Cachexia by Age and Diagnosis Type

While the rate of malnutrition in geriatric patients in the general population was 46%, this rate was found to be 36% in the under-65 age group (*p* < 0.001). Similarly, the prevalence of frailty reached 49% in the geriatric age group, while it was found to be 42% in patients under the age of 65 (*p* < 0.001). The prevalence of cachexia was also higher in geriatric patients at 48%, while it was determined to be 42% in patients under 65 years of age (*p* < 0.001). When examined by diagnosis type, malnutrition, and frailty were most prevalent in upper gastrointestinal cancers (57%, and 52% respectively), while cachexia was most common in hepatopancreatobiliary cancers (58%). Detailed information on the prevalence of malnutrition, frailty, and cachexia by age and diagnosis type is shown in Table 5.

## 4. Discussion

This study fills an important gap in the literature by simultaneously assessing the prevalence of malnutrition, frailty, and cachexia in a large group of patients with different cancer subtypes. The results revealed that these three syndromes were observed at quite high rates in patients undergoing oncological treatment, and that these rates were significantly more frequent in the geriatric age group. These results reveal that cancer and aging together reduce metabolic capacity, resulting in faster development of malnutrition, frailty, and cachexia. The higher prevalence in older patients may be attributed to reduced physiological reserve, chronic inflammation, and age-related muscle loss. Differences across cancer types may be explained by tumor-related metabolic demands and inflammatory burden, particularly in gastrointestinal and lung cancers, which are more strongly associated with cachexia [10,11].

The prevalence of malnutrition in cancer patients has been reported in a wide range both in our study and in the literature. In our study, the prevalence of general malnutrition was found to be 40%, which is consistent with the 41% prevalence reported in the meta-analysis of 65 studies by Jinhui Zhang et al. [12]. When examined by age group, our study found that the prevalence of malnutrition was 36% among patients under 65 years of age, while it rose to 46% among patients aged 65 and over. Similarly, in the NutriCancer study, the prevalence of malnutrition was reported as 44.9% in patients aged 70 and over, and 36.7% in patients under the age of 70 [13]. When evaluated by cancer subtypes, the prevalence of malnutrition in lung cancer patients has been reported to range from 34.5% to 69% in the literature [14]; in our study, the prevalence of malnutrition in lung cancer patients was found to be 45%, corresponding to this range. In our study, the prevalence of malnutrition in patients with colorectal cancer was found to be 35%. In the literature, however, this rate varies significantly between studies, ranging from 23% in the multicenter study by Carl Meissner et al. to 47.8% in the meta-analysis by Hidayet Arifin et al. [15,16]. In our study, the prevalence of malnutrition in breast cancer patients was found to be 23%. Similarly, a study conducted by Xavier Hébuterne et al. found that the prevalence of malnutrition in breast cancer patients was 20.5% [17]. In our study, the prevalence of malnutrition in patients with gynecological cancers was determined to be 45%, while a prospective study conducted in India reported a higher rate of 56.1% [18]. In our study group, the prevalence of malnutrition in upper gastrointestinal cancers was 57%, while Hébuterne et al. reported a higher rate of 60.2% [17]. In our study, the prevalence of malnutrition in patients with hepatopancreatobiliary cancer was found to be 56%. When looking at the literature, a meta-analysis conducted by Jinhui Zhang et al. focusing on pancreatic cancer reports a malnutrition prevalence of 51% [12], while in another study conducted on patients with hepatocellular carcinoma, the prevalence of malnutrition was reported to be between 43% and 54.6% [19]. When these data are considered together, the 53% prevalence in our study is seen to be highly consistent with the literature. In our study, the prevalence of malnutrition in urological cancers was found to be 32%, while the literature indicates that these rates vary between 16.6% and 46.4% [20]. While the prevalence of malnutrition in our head and neck cancer patients is 36%, a multicenter cross-sectional study reported this value as 22.6% [21]. When these data are considered together, it is seen that the prevalence of malnutrition does not show a consistent distribution among cancer patients; rather, it varies widely depending on tumor type, age, population characteristics, and differences in the assessment methods used, and exhibits clinically significant heterogeneity.

In the current study, the prevalence of frailty was determined to be 45% in the population including all cancer subtypes. When evaluated by age group, the prevalence of frailty was 42% in patients under 65 years of age, while this rate rose to 49% in patients aged 65 and over. Although these values are higher than the approximately 42% prevalence of frailty reported in the literature for older cancer patients, they are consistent with existing evidence showing a marked increase in frailty in advanced age [22]. When examined by cancer subtypes, the prevalence of frailty in our lung cancer patients was found to be 47%. Similarly, a systematic meta-analysis reported a prevalence of frailty of 45% in patients with lung cancer [23]. The prevalence of frailty in our colorectal cancer patients is 35%, and similarly, Liwen Xia et al. reported this rate as 31% in colorectal cancer patients in their meta-analysis [24]. The prevalence of frailty in our breast cancer patients was found to be 45%, which is similar to the 43% prevalence rate reported in a systematic review on frailty in older cancer patients [25]. In our study, the prevalence of frailty in patients with gynecological cancer was determined to be 51%. In a systematic review conducted by Di Donato et al. among gynecologic oncology surgery patients, it was reported that the prevalence of frailty reached 60% in some series [26]. In our study, the prevalence of frailty in upper gastrointestinal cancers was 52%, showing a similar trend to the study reporting a frailty prevalence of 52% in older patients with gastrointestinal cancer [27]. In our study, the prevalence of frailty in the hepatopancreatobiliary cancer group was determined to be 44%. Similarly, in a systematic review examining frailty in this patient group, the prevalence of frailty was reported as 39% [28]. In our study, the prevalence of frailty in patients with urological cancer was 36%, showing a similar clinical trend to the prevalence value of 39.7% reported in the literature for patients undergoing major elective urological surgery [29]. The prevalence of frailty in our head and neck cancer patients was found to be 43%, which was similar to the 36% prevalence of frailty reported by Dewansingh et al. in newly diagnosed head and neck cancer patients [30]. When these data are evaluated together, it is seen that frailty varies by cancer subtypes, age, and patient characteristics, but generally remains at remarkably high levels across all malignancy groups.

In the present study, the prevalence of cachexia was found to be 44% in the population including all cancer subtypes. This rate is close to the 52% prevalence of cachexia reported in the NutriAgeCancer study [31]. In terms of age distribution, cachexia was observed in 42% of patients under 65 years of age and 48% of those aged 65 and over, indicating an association with increased risk in older age. In our study group, cachexia was detected in 48% of lung cancer patients; according to a meta-analysis, the prevalence of cachexia in patients with metastatic non-small cell lung cancer was found to be 39%, although individual studies showed that this rate varied between 19% and 63.8% [32]. The prevalence of cachexia in our colorectal cancer patients was found to be 48%. Although the exact same prevalence rate is not found in the literature, some series have reported that the prevalence of cachexia in patients with colorectal cancer reaches 42.7% [33]. In our study group, the prevalence of cachexia in breast cancer patients was 32%, which was found to be consistent with the 30–80% range reported in the literature [34]. The prevalence of cachexia in our upper gastrointestinal cancer group was 45%, and the literature shows that the prevalence of cachexia before treatment in patients with esophageal and gastric cancer was 48% [35]. In our study group, the prevalence of cachexia in patients with gynecological cancer was 47%, which was consistent with the rates reported in the literature according to the Fearon criteria (22.5%) and GLIM criteria (56.3%) [36]. In our patient group, the prevalence of cachexia in patients with hepatopancreatobiliary (HPB) cancer was found to be 58%. This rate is consistent with the high prevalence reported in the literature, particularly regarding pancreatic cancer, and shows a similar trend to the cachexia rates observed in advanced pancreatic tumors, which are 50% at baseline and reach 64% during treatment [37]. However, studies specifically conducted on hepatocellular carcinoma report a prevalence of cachexia ranging from 19% to 34% [38]. Therefore, it can be stated that the 58% prevalence of cachexia detected in our study among patients with hepatopancreatobiliary cancer shows a stronger correlation with the high rates reported in pancreatic cancers; however, it does not fully correspond to the prevalence reported in hepatocellular carcinoma. This difference is thought to stem from the heterogeneity of the patient population in terms of tumor type. The prevalence of cachexia in patients with urological cancer in our patient group was determined to be 35%. This rate was found to be lower than the 44% prevalence reported in the literature for urinary tract malignancies [31]. The prevalence of cachexia in patients with head and neck cancer in our patient group was determined to be 42%, which was consistent with the prevalence of 53.9% reported in large cohorts [31].

Mechanistic interpretation of these findings suggests that malnutrition in cancer patients should not be considered merely a consequence of reduced intake or tumor burden. In hospitalized older adults, the association of MNA and Geriatric Nutrition Risk Index defined nutritional status with leptin, resistin, and oxidative stress markers (Oxidized low-density lipoprotein and glutathione peroxidase) indicates that malnutrition reflects a biologically active state driven by systemic inflammation, adipokine dysregulation, and oxidative stress rather than reduced intake alone [39,40]. These interrelated pathways provide a mechanistic basis for the observed convergence and progressive amplification of malnutrition, frailty, and cachexia in cancer patients.

Our study has some limitations. Due to the cross-sectional design, it is not possible to assess changes over time in malnutrition, frailty, and cachexia, nor their impact on long-term clinical outcomes (treatment response, toxicity, and survival). Furthermore, the absence of prospective follow-up data, functional performance measurements, and biomarker analyses limits the ability to examine the prognostic significance of these conditions in greater detail. Sample heterogeneity among cancer subtypes and differences in diagnostic criteria may limit generalizability in some subgroups. Subgroup analyses, particularly in smaller diagnostic groups, were limited by small sample sizes and reduced statistical power, which may affect the robustness of these findings. In addition, the lack of detailed clinical variables such as disease stage, metastatic burden, treatment intent, and treatment modalities, as well as the use of primarily univariate analyses, may have limited a more comprehensive evaluation of factors associated with malnutrition, frailty, and cachexia. The dichotomization of MNA-SF and FRAIL scale results may have led to a loss of clinically relevant detail. Nevertheless, our study has a strong methodological foundation in that it systematically evaluates the statuses of malnutrition, frailty, and cachexia within the same cohort in a large, single-center cancer population; provides comprehensive prevalence analyses by age and cancer subtypes; and produces results consistent with the literature. In these respects, the study constitutes an important source of data that contributes to the comprehensive management of malnutrition, frailty, and cachexia in clinical practice.

Moreover, emerging artificial intelligence approaches may further enhance the clinical applicability of our findings. Machine learning models integrating multidimensional clinical data may enable early identification of patients at high risk for malnutrition, frailty, and cachexia, while advanced data extraction techniques from electronic health records and imaging may facilitate large-scale and continuous screening. These approaches may support more personalized and dynamic management strategies in oncology care. Recent advances in artificial intelligence, including models such as AlphaFold, further highlight the transformative potential of these approaches in biomedical research [41].

## 5. Conclusions

In this large-scale study, it was demonstrated that malnutrition, frailty, and cachexia have a high prevalence among 4595 patients undergoing active oncological treatment. All these conditions are significantly more common in the geriatric patient group, revealing that the combination of aging and malignancy creates a marked vulnerability in metabolic reserve, muscle strength, functional capacity, and nutritional status.

The prevalence distribution was observed to vary by cancer subtypes; malnutrition, and frailty were most frequently observed in upper gastrointestinal system cancers, while cachexia was most frequently observed in hepatopancreatobiliary cancers. These results show that although catabolic syndromes occur with varying severity depending on the type of cancer, they are generally common in all oncological subtypes.

The results strongly support the need for systematic screening for malnutrition, frailty, and cachexia in routine oncological assessment. Early diagnosis and targeted interventions, particularly in the geriatric age group and in tumors with a high catabolic risk, can improve treatment compliance, reduce complications, and improve clinical outcomes. There is a need for prospective studies to evaluate the effectiveness of intervention strategies to be applied in different cancer subtypes and to develop standard screening protocols.

## Figures and Tables

**Table 1:** Mini Nutritional Assessment—Short (MNA-S) Form.

Description	Score
Has food intake declined over the past 3 months due to loss of appetite, digestive problems, chewing or swallowing difficulties?	0 = Severe decrease in food intake 1 = Moderate decrease in food intake 2 = No decrease in food intake
Weight loss during the last 3 months	0 = Weight loss greater than 3 kg 1 = Does not know 2 = Weight loss between 1 and 3 kg 3 = No weight loss
Mobility	0 = Bed or chair bound 1 = Able to get out of bed/chair but does not go out 2 = Goes out
Have you suffered psychological stress or acute disease in the past 3 months?	0 = Yes 2 = No
Neuropsychological problems	0 = Severe dementia or depression 1 = Mild dementia 2 = No psychological problems
Body mass index (BMI) (weight in kg)/(height in m^2^)	0 = Body mass index less than 19 1 = Body mass index 19 to less than 21 2 = Body mass index 21 to less than 23 3 = Body mass index 23 or greater
Screening score (Total max. 14 points)	12–14 points: Normal nutritional status 8–11 points: At risk of malnutrition 0–7 points: Malnutrition

**Table 2:** Fatigue, Resistance, Ambulation, Illnesses, and Loss of weight (FRAIL) Scale.

Criterion	Description	Score
Fatigue	How much of the time during the past 4 weeks did you feel tired? All of the time = 1, Most of the time = 2, Some of the time = 3, A little of the time = 4, None of the time = 5.	0 = Responses of “3” or “4” or “5” 1 = Responses of “1” or “2”
Resistance	By yourself and not using aids, do you have any difficulty walking up 10 steps without resting?	0 = No 1 = Yes
Ambulation	By yourself and not using aids, do you have any difficulty walking a couple of blocks (e.g., several hundred yards)?	0 = No 1 = Yes
Illness	Did a doctor ever tell you that you have [illness]? How many (see list below): The illnesses include hypertension, diabetes, cancer (other than a minor skin cancer), chronic lung disease, heart attack, congestive heart failure, angina, asthma, arthritis, stroke, and kidney disease.	0 = The total illnesses (0–4) 1 = The total illnesses (5–11)
Loss of Weight	How much do you weigh? Percent weight change is computed as: [[weight 1 year ago − current weight]/weight 1 year ago] * 100.	0 = Percent change <5% 1 = Percent change >5%
Screening score (Total max. 5 points)		0 points: Robust health status 1–2 points: Pre-frail 3–5 points: Frail

**Table 3:** Demographic Characteristics of Patients and Their Distribution by Nutritional, Frailty, and Cachexia Status.

Variable	Category	n	%
Age (years)	Total *	58.9	12.2
	<65 years	2923	64
	≥65 years	1672	36
Sex	Male	2313	50
	Female	2282	50
MNA-SF	8–14 (Non-malnutrition)	2745	60
	0–7 (Malnutrition)	1850	40
FRAIL Scale	0–2 (Non-frail)	2543	55
	3–5 (Frail)	2052	45
Cachexia Status (>5% WL/6 mo or BMI < 20 + >2% WL)	Non-cachectic	2553	56
	Cachectic	2042	44

*: Mean is given instead of “n”; Standard deviation is given instead of “%”; BMI, body mass index; FRAIL Scale, fatigue, resistance, ambulation, illnesses, and loss of weight scale; MNA-SF, mini nutritional assessment—short form; WL, weight loss; mo, months.

**Table 4:** Distribution of Patients by Cancer Group and Included Diagnoses.

Cancer Group	Included Diagnoses (n Per Subgroup)	n
Lung cancer	Non-small cell lung carcinoma	835
	Small cell lung carcinoma	77
Colorectal cancer	Colon carcinoma	660
	Rectal carcinoma	240
Breast cancer	Breast carcinoma	841
Gynecological cancer	Ovarian carcinoma	336
	Endometrial carcinoma	216
	Cervical carcinoma	120
Upper gastrointestinal cancer	Gastric carcinoma	454
	Esophageal carcinoma	127
HPB cancer	Pancreatic carcinoma	222
	Hepatobiliary carcinoma	112
Urological cancer	Bladder carcinoma	107
	Testicular carcinoma	59
	Prostate carcinoma	52
	Renal cell carcinoma	40
Head and neck cancer	Head and neck squamous cell carcinoma	97
Total		4595

HPB, hepatopancreatobiliary.

**Table 5:** Prevalence of Malnutrition, Frailty, and Cachexia in Cancer Patients: Comparison by Age (<65 vs. ≥65 yrs).

Cancer Group	Age Group	N	Malnutrition		Frailty		Cachexia	
			n (%)	*p*	n (%)	*p*	n (%)	*p*
Lung cancer	<65 yrs	480	198 (41)	**0.031**	218 (45)	0.214	224 (47)	0.387
	≥65 yrs	432	209 (48)		214 (50)		214 (50)	
	Total	912	407 (45)		432 (47)		438 (48)	
Colorectal cancer	<65 yrs	557	187 (34)	0.483	188 (34)	0.318	263 (47)	0.664
	≥65 yrs	343	123 (36)		127 (37)		173 (50)	
	Total	900	310 (35)		315 (35)		436 (48)	
Breast cancer	<65 yrs	720	158 (22)	0.487	308 (43)	**0.002**	237 (33)	0.219
	≥65 yrs	121	30 (25)		70 (58)		33 (27)	
	Total	841	188 (23)		378 (45)		270 (32)	
Gynecological cancer	<65 yrs	419	177 (42)	**<0.001**	208 (50)	**0.001**	181 (43)	0.096
	≥65 yrs	253	127 (50)		134 (53)		133 (53)	
	Total	672	304 (45)		342 (51)		314 (47)	
Upper gastrointestinal cancer	<65 yrs	309	163 (53)	0.055	152 (49)	**0.152**	134 (43)	0.372
	≥65 yrs	272	165 (61)		150 (55)		128 (47)	
	Total	581	328 (57)		302 (52)		262 (45)	
HPB cancer	<65 yrs	186	110 (59)	0.239	75 (40)	0.128	105 (57)	0.669
	≥65 yrs	148	78 (53)		72 (49)		87 (59)	
	Total	334	188 (56)		147 (44)		192 (58)	
Urological cancer	<65 yrs	185	67 (36)	0.476	60 (32)	**0.034**	63 (34)	0.812
	≥65 yrs	73	23 (32)		34 (47)		26 (36)	
	Total	258	90 (35)		94 (36)		89 (35)	
Head and neck cancer	<65 yrs	67	17 (25)	**0.001**	22 (33)	**0.002**	31 (46)	0.236
	≥65 yrs	30	18 (60)		20 (67)		10 (33)	
	Total	97	35 (36)		42 (43)		41 (42)	
Total (all cancers)	<65 yrs	2923	1077 (36)	**<0.001**	1231 (42)	**<0.001**	1238 (42)	**<0.001**
	≥65 yrs	1672	773 (46)		821 (49)		804 (48)	
	Total	4595	1850 (40)		2052 (45)		2042 (44)	

Geriatric patients were defined as those aged ≥65 years. HPB, hepatopancreatobiliary; yrs, years.

## Data Availability

The data presented in this study are available on request from the corresponding author.

## Abbreviations

BMI, body mass index; ECOG, Eastern Cooperative Oncology Group; ECOG PS, Eastern Cooperative Oncology Group performance status; FRAIL, Fatigue, Resistance, Ambulation, Illnesses, and Loss of weight; GI, gastrointestinal; HPB, hepatopancreatobiliary; MNA-SF, Mini Nutritional Assessment—Short Form; SD, standard deviation.
